# Predicting overall survival in chordoma patients using machine learning models: a web-app application

**DOI:** 10.1186/s13018-023-04105-9

**Published:** 2023-09-02

**Authors:** Peng Cheng, Xudong Xie, Samuel Knoedler, Bobin Mi, Guohui Liu

**Affiliations:** 1 Department of Orthopedics, Union Hospital, Tongji Medical College, Huazhong University of Science and Technology, 1277# Jiefang Avenue, Wuhan, 430022 Hubei China; 2 Department of Plastic Surgery, Brigham and Women’s Hospital, Harvard Medical School, Boston, MA 02215 USA

**Keywords:** Chordoma, Survival analysis, Machine learning, DeepSurv, Deep learning

## Abstract

**Objective:**

The goal of this study was to evaluate the efficacy of machine learning (ML) techniques in predicting survival for chordoma patients in comparison with the standard Cox proportional hazards (CoxPH) model.

**Methods:**

Using a Surveillance, Epidemiology, and End Results database of consecutive newly diagnosed chordoma cases between January 2000 and December 2018, we created and validated three ML survival models as well as a traditional CoxPH model in this population-based cohort study. Randomly, the dataset was divided into training and validation datasets. Tuning hyperparameters on the training dataset involved a 1000-iteration random search with fivefold cross-validation. Concordance index (C-index), Brier score, and integrated Brier score were used to evaluate the performance of the model. The receiver operating characteristic (ROC) curves, calibration curves, and area under the ROC curves (AUC) were used to assess the reliability of the models by predicting 5- and 10-year survival probabilities.

**Results:**

A total of 724 chordoma patients were divided into training (*n* = 508) and validation (*n* = 216) cohorts. Cox regression identified nine significant prognostic factors (*p* < 0.05). ML models showed superior performance over CoxPH model, with DeepSurv having the highest C-index (0.795) and the best discrimination for 5- and 10-year survival (AUC 0.84 and 0.88). Calibration curves revealed strong correlation between DeepSurv predictions and actual survival. Risk stratification by DeepSurv model effectively discriminated high- and low-risk groups (*p* < 0.01). The optimized DeepSurv model was implemented into a web application for clinical use that can be found at https://hust-chengp-ml-chordoma-app-19rjyr.streamlitapp.com/.

**Conclusion:**

ML algorithms based on time-to-event results are effective in chordoma prediction, with DeepSurv having the best discrimination performance and calibration.

**Supplementary Information:**

The online version contains supplementary material available at 10.1186/s13018-023-04105-9.

## Introduction

A chordoma is an intraosseous tumor that develops from the remnants of the notochord along the nerve axis. It has an incidence of 0.8 per 1 million people and is characterized by sluggish growth, local destruction, low-grade malignancy, and a significant propensity for local recurrence [1, 2]. In the majority (> 95%), the axial skeleton is affected, with approximately equal proportions of the sacrococcygeum, skull base, and active spine being involved [3, 4]. Consequently, the clinical treatment of chordoma is extremely challenging.

Chordoma has a median survival of 7.7 years, with a 5-year survival rate of 72% that drops to 48% and 31% at the 10- and 20-year marks, respectively [2]. Female patients exhibit a slightly superior prognosis, with a median survival of 7.25 years compared to 5.93 years in male counterparts. Additionally, skull base chordomas have a relatively better survival outlook with a median of 6.94 years, in contrast to 5.88 years for chordomas of the mobile spine. It is noteworthy that women and younger patients are more frequently diagnosed with skull base chordomas, and their median survival remarkably extends to approximately 12 years [5].

Complete resection is currently the best course of action for chordoma; however, because the tumor is frequently in close proximity to the brainstem, spinal cord, important nerves, and blood vessels, this is a challenging procedure [6, 7]. According to researchers, aggressive total resection may significantly increase the risk of severe complications and even death. Conversely, incomplete resection increases the recurrence rate [8, 9]. Therefore, surgeons often face difficulty determining surgical options. Chordoma is sensitive to high-dose radiotherapy, which is an important adjuvant therapy for chordoma. However, the therapy can damage surrounding brain tissue, retroperitoneal organ, and spinal cord [3, 8], limiting the use of radiotherapy in the treatment of chordoma. Recent research has demonstrated that high-dose photon/proton radiation improves chordoma patients’ 5-year local control rate by 85%, disease-specific survival by 89%, and long-term failure rate by 20% [9]. Although the safety of the therapy has been improved, it cannot be widely used due to its high cost. Beyond the current standard treatment of complete resection and high-dose radiotherapy for chordoma, research is exploring targeted therapies and immunotherapies, such as PD-1/PD-L1 and CTLA-4 inhibitors [10]. Novel approaches are also being investigated, like those targeting the overexpressed protein brachyury in chordomas [11]. These emerging treatments, although promising, are still in early stages of research and require further studies for validation. Given these situations, the effective evaluation and prediction of the prognostic advantages of patients with chordomas treated in different ways, surgeons are not only guided to optimal treatment strategies, nevertheless, it also allows patients and physicians to develop appropriate individualized treatment plans by weighing various objective factors such as prognosis, risk, income, and economic burden. However, the complex pathological features and treatment modalities of chordoma create challenges in the accurate prediction of chordoma prognosis.

With the purpose of predicting the prognosis of chordoma patients, some prognostic factors associated with chordoma were identified in the previous studies, including tumor size, tumor location, vascular involvement, tumor metastases, and patient age. [12, 13]. Using these factors, nomograms based on Cox proportional hazards (CoxPH) model were commonly constructed to predict survival prognosis. However, the CoxPH model assumes that the variables are linearly related to the outcomes, and its flaw in ignoring nonlinear relationships in the real world is evident.

This issue has now been effectively resolved by machine learning (ML), which is rapidly being employed in oncology, especially for determining the prognosis of bone tumors [14–16]. Unlike the traditional TNM staging and nomogram models based on Cox regression analysis, ML algorithms are capable of capturing complex, nonlinear relationships between variables. This makes them particularly effective in interpreting the inherent complexity and potential nonlinearity present in the data [17]. Numerous ML algorithms for survival analysis have been proposed in prior studies [18, 19] and have demonstrated superior performance compared to traditional Cox algorithms on a variety of medical datasets [17, 20]. Despite individual research efforts in applying ML and survival analysis to chordoma prognosis, a synergistic integration of these two approaches holds promise in unraveling complex interactions among clinicopathological factors impacting patient survival.

In this current study, based on a large cohort database containing chordoma, we developed multiple prognostic models combined with ML targeting chordoma survival outcomes and attempted to compare the differences between ML with traditional learning algorithms. We hypothesize that the ML approach will have superior performance and flexibility compared to traditional Cox regression-based survival models. Finally, the optimal model will be screened by multiple evaluation metrics and developed into an online web calculator for use in clinical practice.

## Materials and methods

### Eligibility criteria and clinical information

**Fig. 1:**
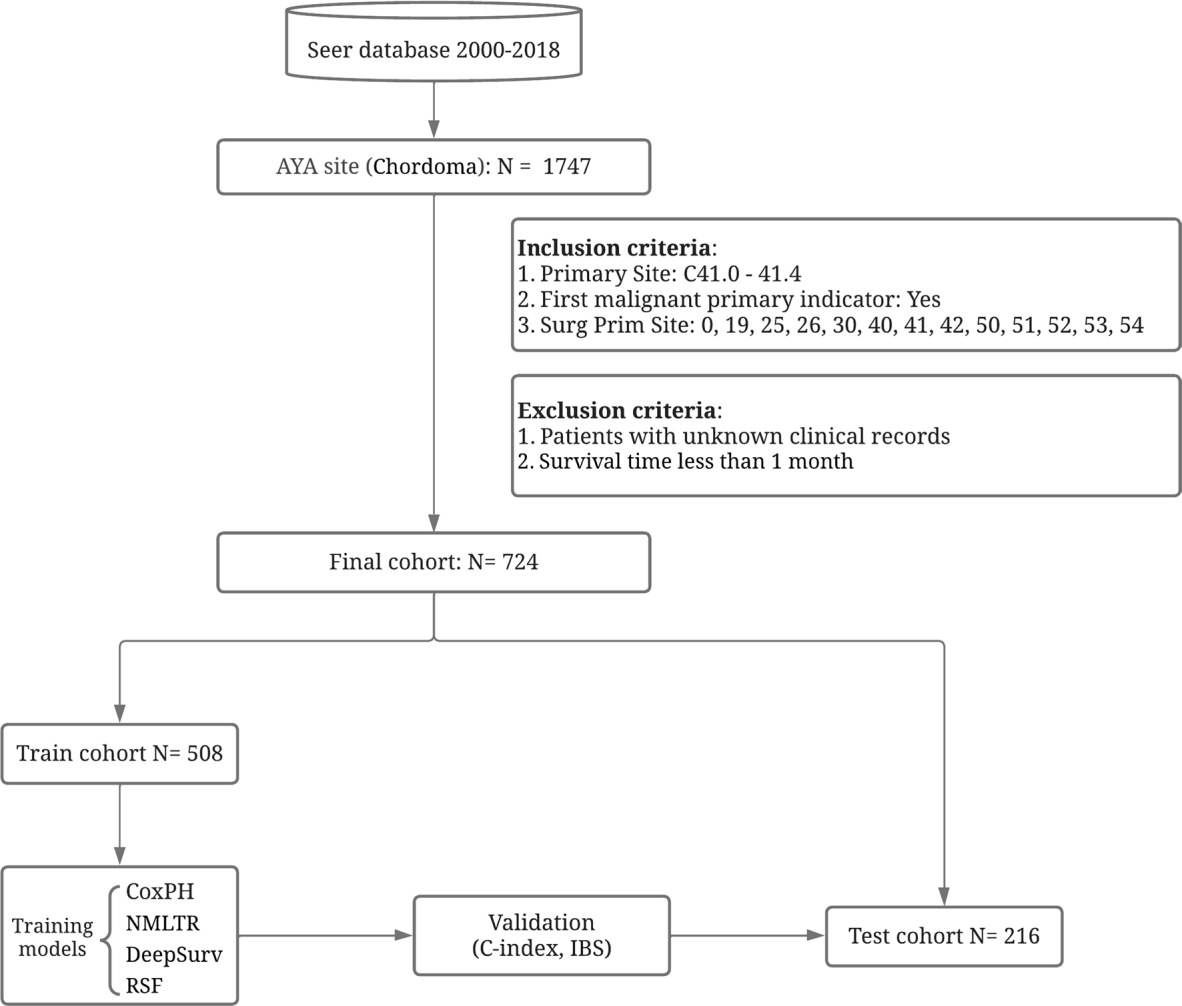
Profile and analytical pipeline of the study

This study used SEER*Stat version 8.3.9 to abstract data. SEER is a National Cancer Institute-funded multi-center, multi-population registry that covers approximately 28% of the US population, with estimated case ascertainment of around 98%. We used data from the SEER Research Plus 18 Registry from 2000 to 2018, with tight inclusion and exclusion criteria. The following were the criteria for inclusion: (1) patients with confirmed chordoma based on the third edition of the International Classification of Diseases for Oncology (ICD-O-3), morphological code (9370-9372), and (2) primary sites with the most common areas associated with the skull, spine, and pelvis (site recode ICD-3/WHO 2008 = 410-414). The following are the exclusion criteria: (1) survival time is unclear (survival time = unknown) and (2) chordoma was not considered as the main tumor (first malignant primary indicator = No). Figure 1 shows the comprehensive selection procedure flowchart.

### Cohort design and model development

The survival endpoint was defined as the (OS) time. Using a 7:3 ratio, the dataset was randomized to training and validation cohorts. The corresponding model was trained on the training set and evaluated on the validation set. For the following variables, 17 clinicopathological factors were extracted from the SEER database: age, sex, marital status, race, histological type, primary site, AJCC T, AJCC N, AJCC M, surgery, radiology, chemotherapy, tumor size, number of tumors, tumor extension, distant metastasis, and median income. We performed univariate and multivariate Cox regression analysis on all 17 prognostic factors and obtained independent prognostic factors (*p* < 0.05). Cox regression, both univariate and multivariate, was used for feature selection. All comparisons were made with a 95% level of confidence, and *p* < 0.05 was considered statistically significant. Then, correlation analyses between features were also conducted to exclude collinear features. For training, three algorithms, including two based on neural networks (DeepSurv, NMLTR) and one on ensemble learning (RSF), were used. Meanwhile, we performed a multivariable CoxPH model for comparative determinations. On the training dataset, hyperparameter tuning was accomplished via a 1000-iteration random search and tenfold cross-validation (Additional file 1: Table S1). The concordance index (C-index) was utilized to assess the performance models in combination with various hyperparameters. The open-source code is available on GitHub at https://github.com/Hust-ChengP/ML_Chordoma, and it provides a full walkthrough of the model-building process and the search space for hyperparameters.

In the development of prognostic models for this study, three advanced ML algorithms were employed, each offering unique capabilities that surpassed traditional Cox regression models. DeepSurv leverages deep learning to extend the Cox proportional hazards model, capturing nonlinear intricacies within the dataset. By using neural networks to model the hazard function, DeepSurv adeptly mapped intricate relationships between covariates and hazard rates, achieving a nuanced understanding of survival patterns [21]. NMLTR, a neural network-based approach tailored for survival analysis, tapped into the representational power of neural networks. This capability allowed NMLTR to efficiently handle high-dimensional or nonlinear data, offering a comprehensive solution to challenges commonly found in medical datasets [22]. RSF stood as an ensemble learning method, extending the traditional random forest to accommodate censored data. Its inherent design facilitated the capture of nonlinearities and interactions between variables without explicit model specification. Coupled with its resilience against overfitting, RSF proved invaluable when analyzing datasets with a mix of categorical and continuous variables. Together, these algorithms exemplified the evolution of survival analysis, enabling a more in-depth and robust exploration of the complexities inherent in chordoma prognosis.

### Model evaluation and validation

The Cox model's predictive power was determined by computing the Harrell C-index, which compared the likelihood of survival to that which was actually experienced. A C-index of 0.5 denoted a random prediction, whereas a C-index of 1.0 denoted a model that predicts events with perfect accuracy. We assessed the significance of the C-index differences using Kang's method [23]. A Brier score, used to evaluate the accuracy of a predicted survival function at a specific time, was also described. It measured the mean square difference between the observed patient state and the anticipated survival probabilities, in which zero was the greatest possible result. A Brier score < 0.25 indicated that the model was valuable. In addition, the integrated Brier score (IBS) gave a calculation of the model's performance across all accessible times. The 5- and 10-year OS was standardized by comparing the predicted survival to the observed survival using a calibration curve. To test the time-dependent sensitivities and the specificities of the model, the receiver operating characteristic (ROC) curves were mapped, and the area under the ROC curves (AUC) value was used for 5- and 10-year survival. Analyzing the net benefits at several probability thresholds, decision curve analysis (DCA) was used to evaluate the model's clinical utility. In addition to the primary metrics previously described, we further augmented our evaluation framework with key discriminative metrics, ensuring a comprehensive assessment of the models. Specifically, sensitivity (or true positive rate) was computed as the ratio of true positives to the sum of true positives and false negatives, providing insights into the model's capability to correctly identify positive cases. Specificity, representing the true negative rate, was determined as the ratio of true negatives to the sum of true negatives and false positives, shedding light on the model's proficiency in correctly classifying negative instances. Accuracy, a holistic measure, was calculated as the proportion of true predictions (both positive and negative) to all predictions, offering a broad perspective on overall model performance. Furthermore, to provide a quantitative measure of calibration, we computed the calibration slope and calibration-in-the-large. The calibration slope gauged the agreement between predicted probabilities and observed outcomes, with a value of 1 indicating perfect calibration. In contrast, calibration-in-the-large measured the average prediction error, with values closer to zero signifying superior calibration.

### Risk stratification

To further validate the viability of our prediction model, we divided all patients into low- and high-risk groups based on the risk scores given by the models. The threshold values corresponded to the mean risk score of each model. After risk stratification, differences between the two groups were compared using survival curves and the log-rank test.

### Feature importance

To evaluate the significance of features to a model, we subsequently replaced each feature value with random values and used the reduction in the model's C-index as the evaluation metric.

### Statistical analysis

All of the clinical data's continuous variables are shown as the mean standard deviation. Categorical variables are described as frequencies and percentages. To compare the variations in the variables between the groups, the Chi-square test and the unpaired two-tailed *t*-test were used. The R programming language was used to perform data pre-processing and visualization (version 4.1.2). ML models were built using the Python programming language's PySurvival module (version 3.6.8).

## Results

### Basic characteristics

Seven hundred twenty-four chordoma patients matched our criteria for inclusion. Table 1 displayed the baseline information of the patients at the time of enrollment. They averaged 53 ± 20 years of age, and 58% of them were male. The training cohort consisted of 508 individuals, while the validation cohort comprised 216 patients. The mean OS for the training group was 79 ± 47 months, and for the validation group, it was 83 ± 49 months. There were no statistically significant differences between the training and validation cohorts for any characteristic (*p* > 0.05) (Table 2).

**Table 1:** Patient demographic, disease, treatment characteristics, and Cox regression analysis

Characteristic	Overall	Univariate Cox			Multivariate Cox		
	*N* = 724^a^	HR	95% CI	*p* value	HR	95% CI	*p* value
Age	53 (20)	1.04	1.04, 1.05	**< 0.001**	1.05	1.04, 1.07	**< 0.001**
Gender							
Female	303 (42%)	–	–		–	–	
Male	421 (58%)	1.07	0.84, 1.36	0.57	1.13	0.81, 1.59	0.47
Marital status							
Not married	321 (44%)	–	–		–	–	
Married	403 (56%)	0.84	0.66, 1.07	0.15	0.81	0.58, 1.13	0.21
Race							
White	607 (85%)	–	–		–	–	
Black	30 (4.2%)	0.26	0.10, 0.69	**0.007**	0.30	0.09, 0.98	**0.047**
Other	78 (11%)	1.00	0.68, 1.48	> 0.99	0.82	0.48, 1.39	0.46
Unknown	9						
Histological type							
Conventional chordoma	672 (93%)	–	–		–	–	
Chondroid chordoma	47 (6.5%)	0.87	0.53, 1.43	0.59	1.07	0.52, 2.19	0.85
Dedifferentiated chordoma	5 (0.7%)	2.82	0.90, 8.82	0.075	1.22	0.23, 6.38	0.81
Primary site							
Bones of skull and face	320 (44%)	–	–		–	–	
Vertebral column	163 (23%)	1.76	1.31, 2.37	**< 0.001**	0.73	0.44, 1.20	0.21
Pelvic bones, sacrum, coccyx	241 (33%)	1.53	1.16, 2.03	**0.003**	0.26	0.15, 0.44	**< 0.001**
AJCC T							
T1	427 (80%)	–	–		–	–	
T2	98 (18%)	2.16	1.56, 2.99	**< 0.001**	1.00	0.52, 1.92	> 0.99
T3	8 (1.5%)	1.85	0.68, 5.00	0.23	1.11	0.29, 4.19	0.88
Unknown	191						
AJCC N							
N0	641 (99%)	–	–		–	–	
N1	7 (1.1%)	2.10	0.87, 5.10	0.10	0.77	0.30, 2.01	0.60
Unknown	76						
AJCC M							
M0	663 (98%)	–	–		–	–	
M1	17 (2.5%)	6.00	3.47, 10.4	**< 0.001**	2.67	0.89, 7.99	0.080
Unknown	44						
Surgery							
None	151 (22%)	–	–		–	–	
Local excision	166 (24%)	0.30	0.21, 0.42	**< 0.001**	0.58	0.33, 1.02	0.059
Partial resection	161 (23%)	0.41	0.29, 0.56	**< 0.001**	0.87	0.51, 1.47	0.60
Radical excision	223 (32%)	0.32	0.23, 0.43	**< 0.001**	0.56	0.34, 0.90	**0.017**
Unknown	23						
Radiotherapy							
Not	412 (57%)	–	–		–	–	
Yes	312 (43%)	0.54	0.42, 0.70	**< 0.001**	0.53	0.35, 0.80	**0.002**
Chemotherapy							
Not	684 (94%)	–	–		–	–	
Yes	40 (5.5%)	2.23	1.48, 3.37	**< 0.001**	0.79	0.39, 1.61	0.52
Tumor size	57 (41)	1.01	1.01, 1.01	**< 0.001**	1.01	1.01, 1.02	**< 0.001**
Unknown	188						
Number of tumors							
1	658 (91%)	–	–		–	–	
> 1	66 (9.1%)	1.40	1.00, 1.98	0.053	1.01	0.62, 1.64	0.96
Tumor extension							
No break in periosteum	119 (18%)	–	–		–	–	
Extension beyond periosteum	501 (75%)	1.49	1.03, 2.15	**0.036**	1.73	1.03, 2.90	**0.038**
Further extension	48 (7.2%)	2.79	1.66, 4.69	**< 0.001**	1.70	0.78, 3.71	0.18
Unknown	56						
Distant metastasis							
Not	663 (98%)	–	–		–	–	
Yes	13 (1.9%)	4.89	2.58, 9.26	**< 0.001**			
Unknown	48						
Median income							
< 60 K	174 (24%)	–	–		–	–	
60–75 K	313 (43%)	0.93	0.70, 1.25	0.65	0.86	0.58, 1.29	0.48
> 75 K	237 (33%)	0.76	0.55, 1.04	0.091	0.91	0.59, 1.39	0.66
Survival months	80 (48)						
Status							
Alive	449 (62%)						
Dead	275 (38%)						

*HR* hazard ratio, *CI* confidence interval

^a^Mean (SD); *n* (%)

Significance of bold is *p*<0.05

**Table 2:** Characteristic distribution of data in training sets and validation sets

	Level	Overall	Train	Validation	*p* value
*n*		724	508	216	
Age [mean (SD)]		53.41 (19.68)	53.12 (19.75)	54.09 (19.56)	0.543
Race (%)	White	607 (84.9)	421 (84.4)	186 (86.1)	0.826
	Black	30 (4.2)	22 (4.4)	8 (3.7)	
	Other	78 (10.9)	56 (11.2)	22 (10.2)	
Primary site (%)	Bones of skull and face	320 (44.2)	217 (42.7)	103 (47.7)	0.440
	Vertebral column	163 (22.5)	119 (23.4)	44 (20.4)	
	Pelvic bones, sacrum, coccyx	241 (33.3)	172 (33.9)	69 (31.9)	
Surgery (%)	None	151 (21.5)	101 (20.5)	50 (24.0)	0.737
	Local excision	166 (23.7)	120 (24.3)	46 (22.1)	
	Partial resection	161 (23.0)	113 (22.9)	48 (23.1)	
	Radical excision	223 (31.8)	159 (32.3)	64 (30.8)	
Radiotherapy (%)	Not	412 (56.9)	290 (57.1)	122 (56.5)	0.945
	Yes	312 (43.1)	218 (42.9)	94 (43.5)	
Chemotherapy (%)	Not	684 (94.5)	479 (94.3)	205 (94.9)	0.877
	Yes	40 (5.5)	29 (5.7)	11 (5.1)	
Tumor size [mean (SD)]		56.80 (40.91)	56.73 (42.56)	56.97 (36.97)	0.951
Tumor extension (%)	No break in periosteum	119 (17.8)	81 (17.2)	38 (19.3)	0.666
	Extension beyond periosteum	501 (75.0)	354 (75.2)	147 (74.6)	
	Further extension	48 (7.2)	36 (7.6)	12 (6.1)	
Distant metastasis (%)	Not	663 (98.1)	463 (97.9)	200 (98.5)	0.805
	Yes	13 (1.9)	10 (2.1)	3 (1.5)	
Survival months [mean (SD)]		80.12 (47.91)	78.95 (47.38)	82.87 (49.15)	0.314
Status (%)	Alive	449 (62.0)	320 (63.0)	129 (59.7)	0.456
	Dead	275 (38.0)	188 (37.0)	87 (40.3)	

### Feature selection

**Fig. 2:**
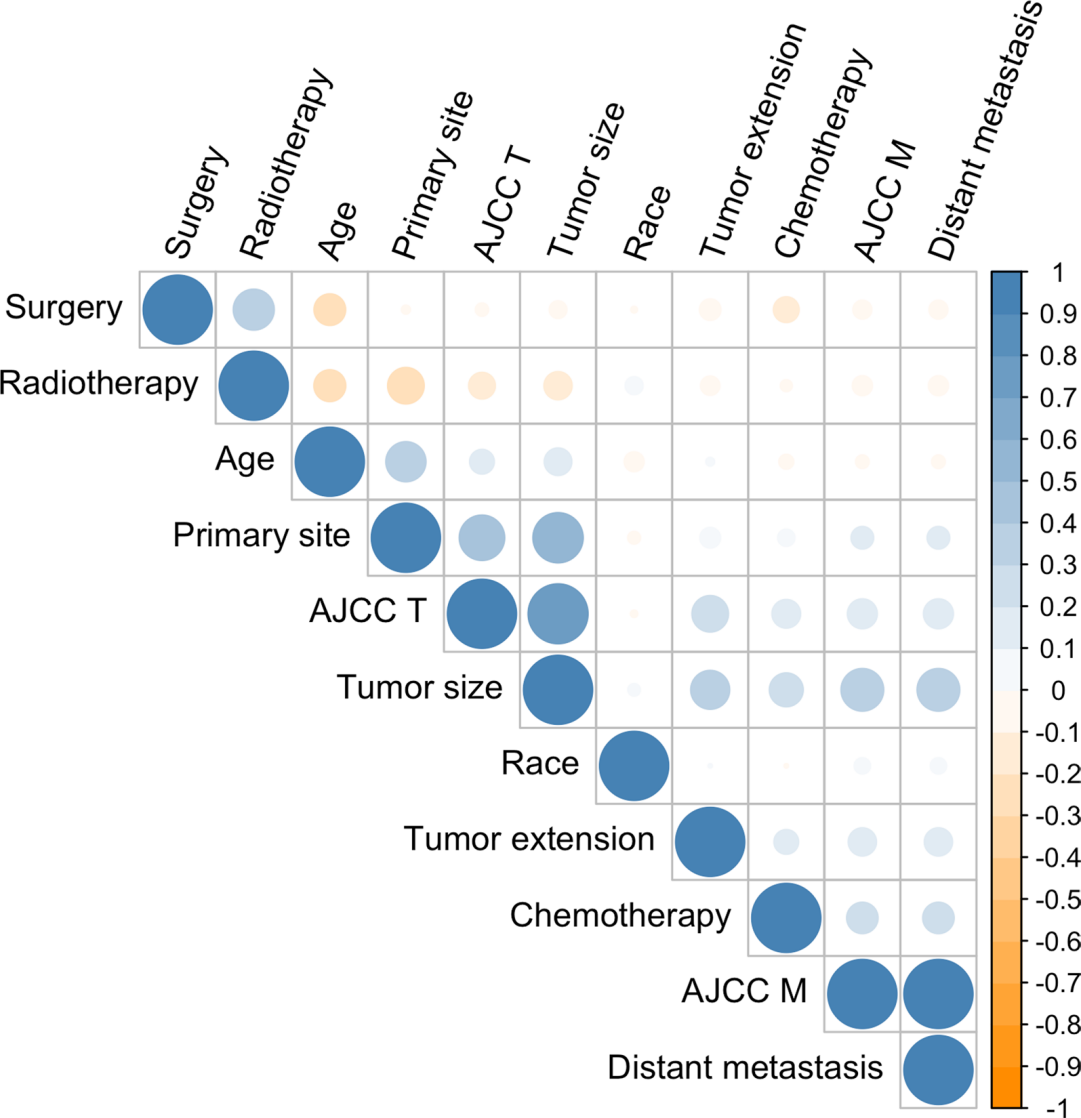
Coefficients of correlation for every pair of variables in the dataset. The calculated correlation values are evenly distributed between − 1 and + 1. The strength of a negative or positive correlation increases as a value approaches one of the two final values

**Fig. 3:**
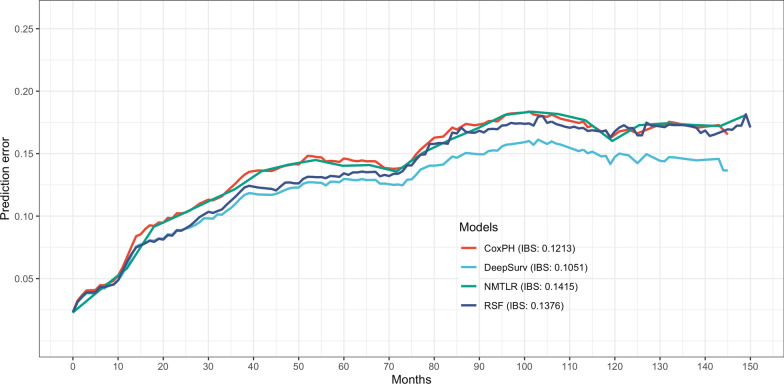
Prediction error curve. As a guideline, a meaning model should have a prediction error of less than 0.25

All of the data were subjected to univariate and multivariate analysis of the Cox regression model. Table 2 displayed the results of the Cox regression analysis, which selected 11 important parameters as predictors of survival (age, race, primary site, AJCC T, AJCC M, surgery, radiotherapy, chemotherapy, tumor size, tumor extension, and distant metastasis). In the correlation analysis, AJCC T and AJCC M features were omitted due to their significant collinearity with other features (Fig. 2). Nine variables were ultimately identified as independent prognostic factors and used in the development of the final model (Fig. 3).

### Model comparisons

**Fig. 4:**
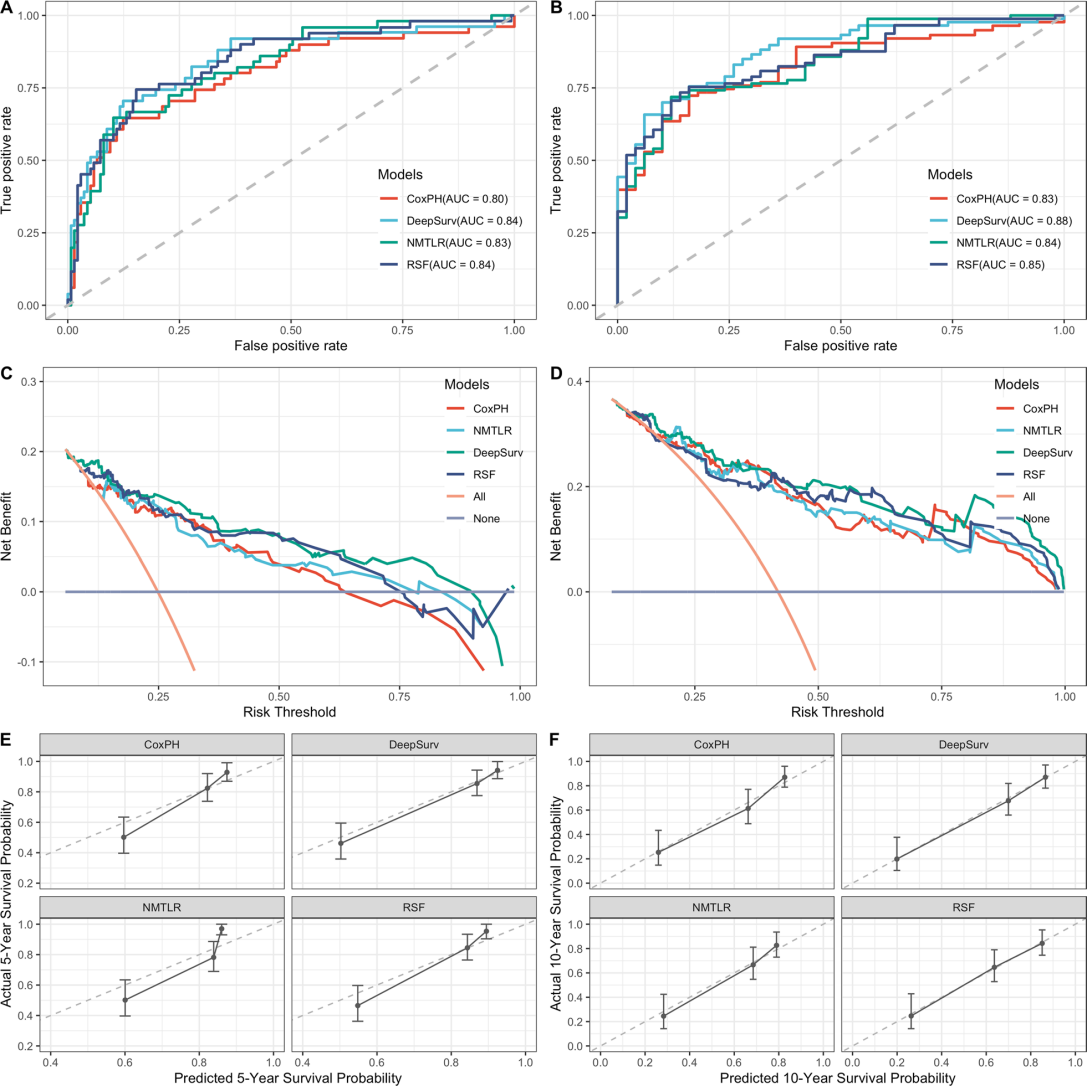
The receiver operating curves (ROC), decision curve analysis (DCA), and calibration curves for 5- and 10-year survival predictions. ROC curves for **A** 5- and **B** 10-year survival predictions. DCA for **C** 5- and **D** 10-year survival predictions. Calibration curves for **E** 5- and **F** 10-year survival predictions

Table 3 presented the results of a comparison between the ML models and the CoxPH model in terms of their predictive ability. In the validation dataset, the three ML models significantly outperformed the standard CoxPH (C-index: 0.735) in terms of C-index (DeepSurv: 0.795, *p* < 0.05; NMLTR: 0.745, *p* < 0.05; RSF: 0.758, *p* < 0.05), with DeepSurv having the highest C-index of the three. The models were not overfitting, as evidenced by the low variation in the C-index obtained from the training and validation data sets (DeepSurv: 0.804; NMLTR: 0.768; RSF: 0.792; CoxPH: 0.754). IBS values for four models were 0.105 for DeepSurv, 0.142 for NMTLR, 0.138 for RSF, and 0.121 for CoxPH. Figure 4A and B graphically presented the discriminative capabilities of the ML models in predicting 5-year and 10-year OS on the validation datasets. Across both time intervals, the ML models exhibited superior discriminatory power compared to the traditional CoxPH model. The AUC values for these models were 0.83–0.84 for the 5-year prediction and 0.84–0.88 for the 10-year prediction, while the CoxPH model yielded AUC values of 0.80 for the 5-year prediction and 0.74 for the 10-year prediction. Moreover, the sensitivity, specificity, and accuracy metrics extracted from Table 3 further highlighted the enhanced performance of the ML models over the CoxPH model. Notably, the ML models consistently outperformed in terms of calibration, as evidenced by the calibration slope and calibration-in-the-large metrics, underscoring their reliability in survival probability estimation. As illustrated in Fig. 4C and D, the clinical utility of our models was further evaluated using DCA. These graphs demonstrate that decisions made using ML models were much superior to those made with the CoxPH model for clinically relevant thresholds. Overall, among these models, the DeepSurv model produced the best results. There was a strong correlation between model-based and Kaplan–Meier estimations of survival time, as shown by the calibration curves for both 5- and 10-year survival probabilities (Fig. 4E, F).

**Table 3:** Performance of four survival models

	C-index		*P* value	IBS	5-year						10-year					
	Train^a^	Test^a^			AUC	SEN	SPE	ACC	CS	CITL	AUC	SEN	SPE	ACC	CS	CITL
CoxPH	0.754	0.735	Ref	0.121	0.801	0.785	0.784	0.794	1.338	− 0.052	0.832	0.774	0.673	0.746	1.253	0.140
NMLTR	0.768	0.745	**0.033**	0.142	0.832	0.810	0.765	0.783	1.718	− 0.089	0.844	0.883	0.635	0.815	1.163	0.082
DeepSurv	0.804	0.795	**< 0.001**	0.105	0.843	0.844	0.756	0.804	1.223	− 0.022	0.884	0.766	0.731	0.757	1.102	0.106
RSF	0.792	0.758	**0.021**	0.138	0.838	0.765	0.764	0.804	1.317	− 0.056	0.847	0.891	0.654	0.825	1.132	0.114

*CoxPH* standard cox proportional hazards, *NMLTR* neural multi-task logistic regression, *RSF* random survival forest, *IBS* integrated brier score, *SEN* sensitivity, *SPE* specificity, *ACC* accuracy, *CS* calibration slope, *CITL* calibration in the large

^a^C-index in train and test dataset were calculated separately, the other three metrics were calculated in the test set

### Risk stratification

**Fig. 5:**
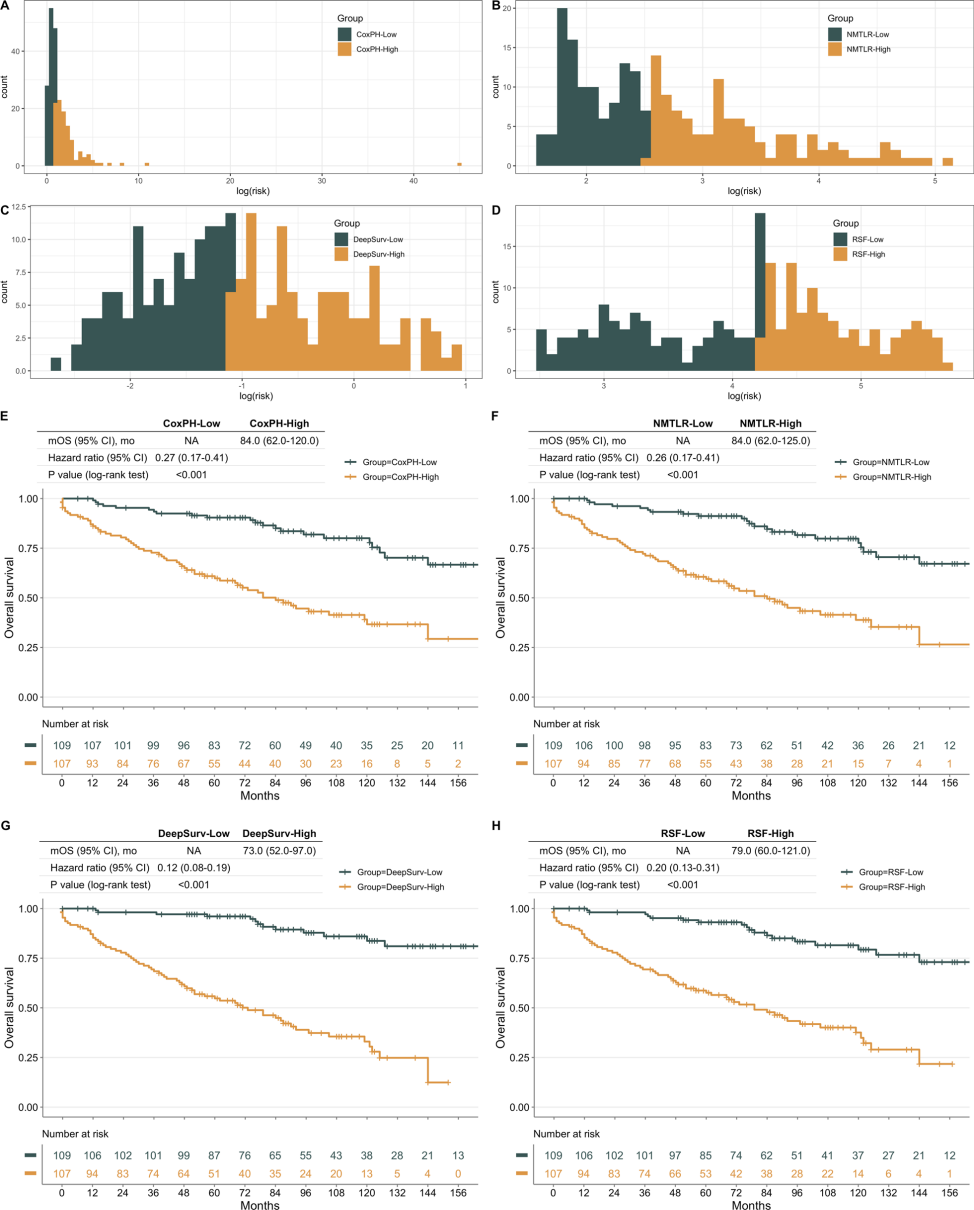
Developed models produce Kaplan–Meier (KM) curves for chordoma patients with varying risk levels. **A**–**D** Based on the median risk value given by the model for all patients, the patients were separated into high-risk and low-risk groups. The KM curves of patients grouped by various models are depicted in (**E**–**H**), respectively

As depicted in Fig. 5, all models performed well when defining high-risk groups, patients assigned to the low-risk group had a short survival time (log-rank test *p* < 0.01). Of all the models, the two groups stratified by the DeepSurv model showed the most significant differences, in which high-risk patients exhibited lower median survival time and a higher risk of death compared to low-risk patients.

### Feature importance

**Fig. 6:**
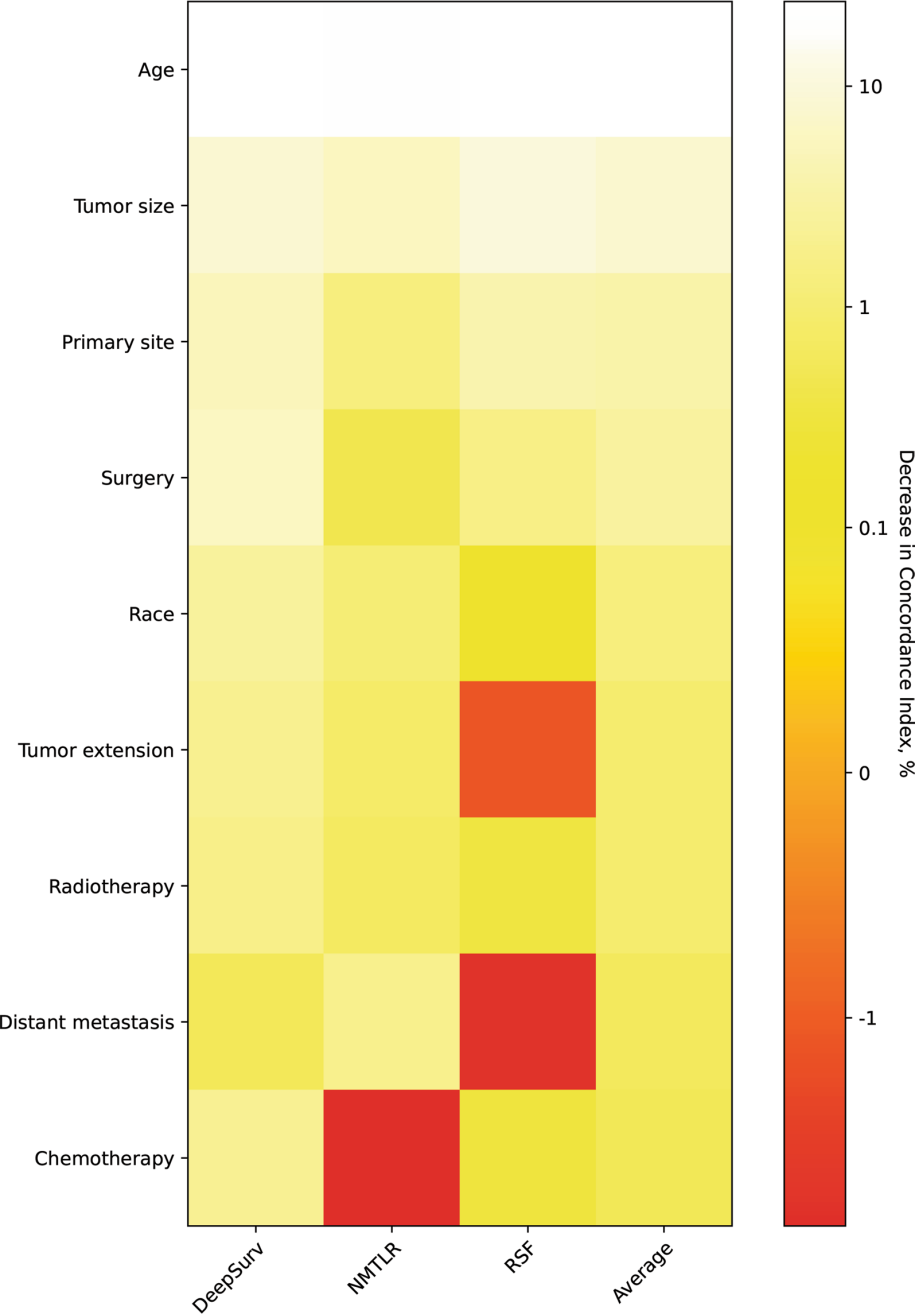
Heatmap depicting the significance of features for DeepSurv, neural network multi-task logistic regression (NMLTR), and random survival forest (RSF) models

The C-index reduction of each feature after random value substitution (Fig. 6) revealed features that are critical to model accuracy for prognosis. Five out of nine factors, including age, tumor size, primary site, surgery, and race, contributed to an average 1% drop in the C-index.

### Algorithm deployment

Based on the optimal performance DeepSurv model, we developed a user-friendly web application for prediction, accessible at https://hust-chengp-ml-chordoma-app-19rjyr.streamlitapp.com/.

## Discussion

Accurate prediction of survival outcomes for patients with chordoma is essential for patient counseling, follow-up, and decision making on treatment options. Some of the factors that influence survival time in patients with chordoma include age, tumor size, histological type, tumor grade, and metastasis have been widely reported [7, 24, 25]. As research into chordoma continues, more and more prognostic factors such as imaging [26, 27], genetics [28, 29], and biomarkers [30, 31] have been explored for use in the survival analysis of chordoma patients. The limitations of linear relational models based on the traditional CoxPH model have become increasingly apparent in the face of the massive amount of multidimensional data [32]. This issue has a good solution in ML, which has begun to be studied and used in a number of medical sectors. As a result, we created three ML models and evaluated their effectiveness against the traditional CoxPH model to predict the survival possibility of chordoma patients.

In the current study, four models for predicting chordoma patient survival, namely DeepSurv, NMTLR, RSF and CoxPH, were constructed and compared by collecting and analyzing potentially significant characteristics of 724 patients with chordoma from the SEER database. We performed a Cox proportional risk regression analysis on all included patients with chordoma to identify prognostic risk factors, including age, race, primary site, AJCC T, AJCC M, surgery, radiotherapy, chemotherapy, tumor size, tumor extension, and distant metastasis, which were consistent with previous reports in the literature [7]. A comparison of several models revealed that the DeepSurv prediction model developed by Katzman et al. performed best, followed by RSF, NMTLR and CoxPH. The DeepSurv model had a C-index of 0.804 and 0.795 for the training and validation cohorts, respectively, and demonstrated an improvement in model accuracy, clinical benefits, and calibration. In addition, we have integrated the best-performing DeepSurv model into a user-friendly web-based application that can be accessed using the following link: https://hust-chengp-ml-chordoma-app-19rjyr.streamlitapp.com/.

There have been some previous studies on prognostic models for chordoma, most of which are based on traditional Cox regression analysis. For example, researchers Lin et al. [33] applied a nomogram to predict OS for spine chordoma with a C-index of 0.73 in the validation dataset. Meng et al. [34] constructed a nomogram model to predict the prognosis of chordoma based on a multi-center database incorporating preoperative and postoperative clinical information on patients. Although their model achieved a C-index of 0.76 on the validation set, the sample size was only 276, which limited the reliability of the model. But our established DeepSurv prediction model achieved a C-index of 0.78 and its 3- and 5-year AUCs were 0.82 and 0.84, respectively, with the results superior to those previously reported in the literature, reflecting the advantages of DeepSurv in the analysis of survival prediction data.

The application of DeepSurv in predicting tumor survival offered a paradigm shift in the realm of oncological prognostication. At its core, DeepSurv was adept at capturing nonlinear relationships between predictors and outcomes, mirroring the intricate and multifaceted nature of real-world clinical scenarios [35]. This nonlinearity stood in stark contrast to traditional models, which frequently relied on linear relational analyses and might not fully encapsulate the complexities inherent to oncological data. Moreover, the versatility of deep learning, as exemplified by DeepSurv, extended beyond mere data fitting. It paved the way for a more holistic integration of diverse data types, from imaging to genetic markers, and potentially harnessing the power of multimodal information fusion techniques [36]. As the field of oncology continues to evolve, with an ever-expanding repository of tumor mechanisms and biomarkers, the potential for integrated big data analyses to refine and enhance survival predictions becomes increasingly evident [37]. Notably, our study's contribution transcends the theoretical realm. By embedding our DeepSurv model into an intuitive web application, we bridge the gap between advanced computational research and clinical practice. This digital platform, readily accessible to healthcare professionals globally, stands as a testament to the translational prowess of our research. It promises to revolutionize patient interactions, offering clinicians a robust tool to aid in patient counseling, inform therapeutic strategies, and optimize follow-up regimens.

In the broader academic landscape, our findings resonate with emerging literature on the utility of ML in oncological prognostication. For instance, a study on non-metastatic chondrosarcoma patients highlighted the potential of ML models in enhancing clinical decision making [15]. Similarly, the application of ML in predicting survival outcomes for spinal and pelvic Ewing's sarcoma underscored the adaptability of these models across diverse cancer subtypes [38]. These studies, in tandem with our findings, underscored the transformative potential of in oncology, heralding a new era of data-driven, personalized patient care.

The five features driving results in our model (age, tumor size, primary site, surgery, and race) aligned with established prognostic factors reported in the literature. Specifically, age is a critical prognostic factor, as chordomas rarely affect patients under 40, with a peak incidence between 50 and 60 years old [5]. Older age at diagnosis confers worse OS. Tumor size also correlates with prognosis, as larger tumors at presentation lead to more local destruction and compression of surrounding tissues, causing worse symptoms and requiring more aggressive surgery. Tumor location is relevant, with skull base chordomas having better prognoses than sacral or spinal tumors, possibly due to earlier diagnosis from symptoms and improved surgical accessibility. Complete surgical resection is a major predictor of prognosis, but is often limited by anatomical constraints. Positive margins after surgery increase risks of recurrence and death. Finally, race may play a role, as chordomas have a higher incidence in Caucasian versus African-American populations, implying potential genetic factors [5]. Overall, the features driving our model are consistent with clinical factors known to impact chordoma outcomes.

While our study leveraged advanced ML models to predict survival outcomes in chordoma patients, it was essential to acknowledge the inherent limitations of these models. ML models, especially deep learning architectures, required large datasets to train effectively. The risk of overfitting, where the model performed exceptionally well on the training data but poorly on unseen data, was a known challenge [18]. Additionally, while these models could capture nonlinear relationships in the data, their interpretability rema﻿ined a concern, making it difficult to understand the underlying reasons for specific predictions [19]. The absence of external validation across diverse geographies and ethnic groups further limited the generalizability of our findings. Moreover, ML models were sensitive to the quality and completeness of the input data. The SEER database, while comprehensive, might lack granular details that could influence survival outcomes, potentially introducing biases. As with all predictive models, continuous validation and updating were crucial to maintain their accuracy and relevance in the face of evolving clinical practices and patient populations.

## Conclusion

Our study successfully employed ML models, particularly the DeepSurv model, to predict OS in chordoma patients. The DeepSurv model outperformed traditional CoxPH models, demonstrating the potential of ML in enhancing predictive accuracy in the medical field. The significant prognostic factors identified, such as age, tumor size, primary site, surgery, and race, align with existing literature, reinforcing their clinical relevance. The development of a user-friendly web application further emphasizes the practical applicability of our findings. Future research directions could focus on integrating more comprehensive clinical datasets, including radiation modality, dosing, and pre/postoperative functional scores. Additionally, exploring the potential of radiomics, as suggested by recent studies, could further refine prediction models. Collaborative efforts across multiple centers and geographies would also enhance the external validity of these models, ensuring their broader applicability.

### Supplementary Information

**Table S1.** Hyperparameters and their search ranges for machine learning models in chordoma survival prediction.

## Data Availability

The data that support the findings of this study are available from the corresponding author upon reasonable request.
