# Supplementary material for: Predicting overall survival in chordoma patients using machine learning models: a web-app application
Source: J Orthop Surg Res. 2023 Sep 2;18:652. doi: 10.1186/s13018-023-04105-9 (PMC10474690; doi:10.1186/s13018-023-04105-9)
Supplement: Supplementary file 1 — Table S1. Hyperparameters and their search ranges for machine learning models in chordoma survival prediction. [file 13018_2023_4105_MOESM1_ESM.docx]

Table S1. Hyperparameters and Their Search Ranges for Machine Learning Models in Chordoma Survival Prediction.

| **Model** | **Hyperparameter** | **Values** | **Explanation** |
| --- | --- | --- | --- |
| NMTLR | structure - num_layers | 1 to 5 with a step of 1 | Number of hidden layers in the neural network. |
| NMTLR | structure - num_units | 8 to 99 with a step of 1 | Number of neurons in each hidden layer. |
| NMTLR | structure - activations | ['Atan', 'BentIdentity', 'BipolarSigmoid', 'CosReLU', 'Gaussian', 'Hardtanh', 'InverseSqrt', 'LeakyReLU', 'LeCunTanh', 'LogLog', 'LogSigmoid', 'ReLU', 'SELU', 'Sigmoid', 'Sinc', 'SinReLU', 'Softmax', 'Softplus', 'Softsign', 'Swish', 'Tanh'] | Activation function used in each hidden layer. |
| NMTLR | optimizer | ['adadelta', 'adagrad', 'adam', 'adamax', 'rmsprop', 'sgd'] | The optimization algorithm used for training the model. |
| NMTLR | bins | 10 to 99 with a step of 1 | Number of intervals to discretize the survival times. |
| NMTLR | lr | 1e-05 to 0.001 with an increment of 1e-05 | Learning rate, which controls the update magnitude of the model parameters during each iteration. |
| NMTLR | num_epochs | 50 to 1000 with a step of 1 | Number of iterations for training the model. |
| NMTLR | dropout | [0.1, 0.2, 0.3, 0.4] | Regularization technique that randomly sets input units to 0 to prevent overfitting. |
| NMTLR | l2_reg | 0.0001 to 0.01 with an increment of 0.0001 | Coefficient for L2 regularization. |
| NMTLR | l2_smooth | 0.0001 to 0.01 with an increment of 0.0001 | Coefficient for smooth L2 regularization. |
| NMTLR | batch_normalization | False to True with a step of 1 | Indicates whether batch normalization is used after each hidden layer. |
| DeepSurv | structure - num_layers | 1 to 5 with a step of 1 | Number of hidden layers in the neural network. |
| DeepSurv | structure - num_units | 8 to 99 with a step of 1 | Number of neurons in each hidden layer. |
| DeepSurv | structure - activations | ['Atan', 'BentIdentity', 'BipolarSigmoid', 'CosReLU', 'Gaussian', 'Hardtanh', 'InverseSqrt', 'LeakyReLU', 'LeCunTanh', 'LogLog', 'LogSigmoid', 'ReLU', 'SELU', 'Sigmoid', 'Sinc', 'SinReLU', 'Softmax', 'Softplus', 'Softsign', 'Swish', 'Tanh'] | Activation function used in each hidden layer. |
| DeepSurv | optimizer | ['adadelta', 'adagrad', 'adam', 'adamax', 'rmsprop', 'sgd'] | The optimization algorithm used for training the model. |
| DeepSurv | lr | 1e-05 to 0.001 with an increment of 1e-05 | Learning rate, which controls the update magnitude of the model parameters during each iteration. |
| DeepSurv | num_epochs | 50 to 5000 with a step of 1 | Number of iterations for training the model. |
| DeepSurv | dropout | [0.1, 0.2, 0.3, 0.4] | Regularization technique that randomly sets input units to 0 to prevent overfitting. |
| DeepSurv | l2_reg | 0.0001 to 0.01 with an increment of 0.0001 | Coefficient for L2 regularization. |
| DeepSurv | batch_normalization | False to True with a step of 1 | Indicates whether batch normalization is used after each hidden layer. |
| RSF | num_trees | 20 to 1000 with a step of 1 | Number of trees to be built in the forest. |
| RSF | max_features | ['sqrt', 'log2', 'all', 0.1, 0.2] | Number of features to consider for the best split. |
| RSF | min_node_size | 5 to 80 with a step of 1 | Minimum number of samples required in a leaf node. |
| RSF | sample_size_pct | [0.2, 0.4, 0.6, 0.8] | Percentage of sample size for each tree. |
| RSF | importance_mode | ['impurity', 'impurity_corrected', 'permutation', 'normalized_permutation'] | Method to compute the feature importance. |
